# Common Food Sources for Macrobenthic Communities in Different Bottom Covers: Submerged Macrophytes and Benthic Cyanobacteria in a Japanese Temperate Lake

**DOI:** 10.1002/ece3.70359

**Published:** 2024-11-13

**Authors:** Kenzi Takamura, Natsuko I. Kondo, Nobuyoshi Nakajima

**Affiliations:** ^1^ National Institute for Environmental Studies Tsukuba Ibaraki Japan

**Keywords:** benthic crustacean, Chironomid, *Microseira wollei*, stable‐isotope analysis, submerged macrophyte

## Abstract

Macrobenthic communities in a lake are affected by the type of bottom cover such as macrophytes or algae. In the southern basin of Lake Biwa, mats of the benthic cyanobacteria (BC) *Microseira wollei* widely cover the lake bottom and are interspersed with submerged macrophytes (SMs). Because different macrobenthos species appear to occur at those bottoms, we investigated the composition of the communities. Furthermore, as *M. wollei* is supposed to be inedible to consumer organisms owing to its hard sheath and toxins, the food possibility of the cyanobacteria and macrophytes for macrobenthos was analyzed. In this study, macrobenthos were collected, identified molecularly, counted in the BC and SM zones, and analyzed for carbon and nitrogen stable‐isotopic compositions. In the BC zone, asellids and amphipods dominated the macrobenthic community, while chironomids dominated the SM zone. The stable‐isotope analysis revealed that *M. wollei* was a minor food source for macrobenthos and other higher‐level consumers, with some macrophytes, particulate organic matter and bottom sediment potentially being a major source. The dominance of crustacean macrobenthos in *M. wollei* mats suggested that they provided a refuge from predation for crustaceans, whereas SMs were not sufficiently abundant to achieve this. Although different macrobenthic communities in the BC and SM zones were likely supported by common food sources, with the exeption of *M. wollei*, the present study was conducted over a short period and lacked advanced methods for gut content analysis. Therefore, further monitoring and food web analysis are required.

## Graphical abstract

A variety of macrobenthic invertebrates were found in the benthic cyanobacterial and the macrophyte zones in the southern basin of Lake Biwa. Their compositions were clearly different between the zones, but relied on common food sources.

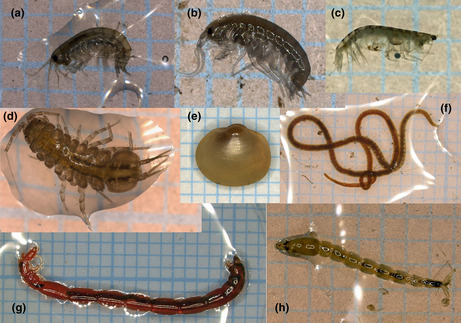

## 1 Introduction

Lakes under a high level of anthropological stress such as eutrophication are often characterized by massive blooms of phytoplankton or by dense growth of submerged plants. Meanwhile, primary producers of a different type also dominate these lakes. These include benthic filamentous algae, green algae, and cyanobacteria (Vadeboncoeur et al. 2021). At the bottom of the southern basin of Lake Biwa, the largest lake on the Japanese Isles, benthic filamentous algae spatially alternate with submerged plants (Haga, Sakai, and Ishikawa 2019), and the predominant taxon is the cyanobacterium *Microseira wollei* (basionym *Lyngbya wollei*; McGregor and Sendall 2015), which is found in all seasons. It grows attached to muddy sediments or bottom structures such as macrophytes and forms amorphous mats with loosely tangled filaments only on the lake bottom, although mats form at the water surface, in the water column, and at the bottom in North American freshwaters (Speziale, Turner, and Dyck 1991). This cyanobacterium has drawn attention from the viewpoint of ecosystem management, not only in North America (Hudon, Sève, and Cattaneo 2014; Speziale, Turner, and Dyck 1991) but also in East Asia (Bae, Kang, and Park 2020; Haga, Sakai, and Ishikawa 2019), while no recorded outbreaks have occurred in Australia (McGregor and Sendall 2015). In North American and East Asian waters, it forms dark mats at the bottom and/or in the water column and causes inconvenience, such as an unpleasant taste and odor in the water and toxin production (Hudon, Sève, and Cattaneo 2014). Because the mats are a refuge for a certain type of benthic invertebrates, low fish production occurs because of the low availability of invertebrates as food (Hudon et al. 2012). Furthermore, there is a strong possibility that *M. wollei* itself is not consumed efficiently by consumer organisms such as benthic macroinvertebrates (macrobenthos). However, there has been controversy regarding this idea (Hudon et al. 2012; Lévesque, Cattaneo, and Hudon 2015), and actually, a certain group of macrobenthos has been collected exclusively from the mats during the Haga, Sakai, and Ishikawa (2019) survey of macrophytes by one of the authors (Takamura 2022).

However, cyanobacterial blooms are not always inedible to invertebrates. The planktonic cyanobacterium *Microcystis aeruginosa* is a primary producer that blooms in eutrophic waters. Negative effects of this cyanobacterium on higher trophic levels, such as zooplankton, occur through microcystin toxicity and feeding interference in which large *Microcystis* colonies are hard to ingest (Krevš, Koreivienė, and Mažeikaitė 2010; Lehman et al. 2010). However, the negative effects of toxins on zooplankton feeding appear to decrease through decomposition (Hanazato and Yasuno 1987a, 1987b), and benthic chironomids may consume decomposed cyanobacteria during the cool season (Iwakuma and Yasuno 1987). This line of research has been developed recently using monitoring data and biochemical, isotopic, or molecular analyses of food webs in freshwater and the sea. From the monitoring data, negative effects of cyanobacteria on phytoplankton and zooplankton were not found in the Baltic Sea (Suikkanen et al. 2021), but strong positive relationships between cyanobacterial concentrations and the biomass of several herbivorous zooplankton taxa were found in Lake Erie (Briland et al. 2020). Cyanobacterial fatty acids and amino acids may be incorporated through microbial loop by mesozooplankton (Eglite et al. 2019). Furthermore, DNA metabarcoding supports the idea that cyanobacteria are the main source of primary production in pelagic food webs (Novotny et al. 2023).

Meanwhile, aquatic plants offer food and habitats for other members of lake ecosystems (Bakker et al. 2016; do Nascimento Filho, Gama, and do Nascimento Moura 2021; Walker, Wijnhoven, and van der Velde 2013; Wood et al. 2017). They are often dominant in eutrophic lakes. In particular, submerged plants grow densely even in the offshore parts of shallow lakes and switch dominance with phytoplankton, which is triggered by physiological, chemical, or biological disturbances (Hobbs et al. 2016; Scheffer et al. 1993, 2001; Zimmer et al. 2016). They generate problems in various types of ecosystem services such as boat traffic, fishing, swimming, and hydrodynamics (Verhofstad and Bakker 2019) but provide microscale habitats for algae, microorganisms, and macroinvertebrates with periphytic and planktonic habits. Plants and their periphyton are generally edible to benthic invertebrates and fish, forming a base for fertile ecosystems, although the extent of herbivory on aquatic plants has been debated (Bakker et al. 2016).

Lake Biwa consists of two major basins: the southern and northern basins. The southern basin is approximately 4 m deep on average and much shallower than the northern part (approximately 41 m on average). The basin is characterized by dense growth of submerged macrophytes (SMs) or phytoplankton blooms during the warm season (Ishikawa et al. 2019). *Microseira wollei* has recently become dominant at the lake bottom where the abundance of SMs is low (Haga, Sakai, and Ishikawa 2019); however, the reason for this increase is unclear and its origin has not been identified. This lake is a major source of water for the Kinki region, which is a heavily populated area in Japan. The lake is also rich in endemic species owing to its unique geological history of isolation and orogenic movement (Inoue, Kobayashi, and Nishino 2020). The fauna and abundance of macrobenthos were reported from 1966 to 1973 (Mori 1978), and the relationship between the abundance of macrophytes and macrobenthos has been studied (Ishikawa, Inoue, and Hamabata 2020). However, macrobenthic taxa have not been fully identified at the species level. Therefore, more detailed species identification methods are needed to study macrobenthic communities.

The focus of this study was to determine whether these different bottom cover types (benthic cyanobacteria (BC) and submerged plants) are inhabited by different macrobenthos and to nutritionally support them. We collected macrobenthos samples, compared their abundance and composition, and analyzed the organic material flow from primary producers. We used DNA barcoding and molecular species delimitation to identify macrobenthos. Measurements of δ^13^C and δ^15^N stable isotopes for the analysis of the food web were also performed. That is, this study was comprised of two parts: macrobenthos composition and isotope analysis. We report contrasting macrobenthic communities but common food sources at the bottom of the southern basin of Lake Biwa.

## 2 Materials and Methods

### 2.1 Study Sites and Quantitative Sampling of Macrobenthos

**FIGURE 1:**
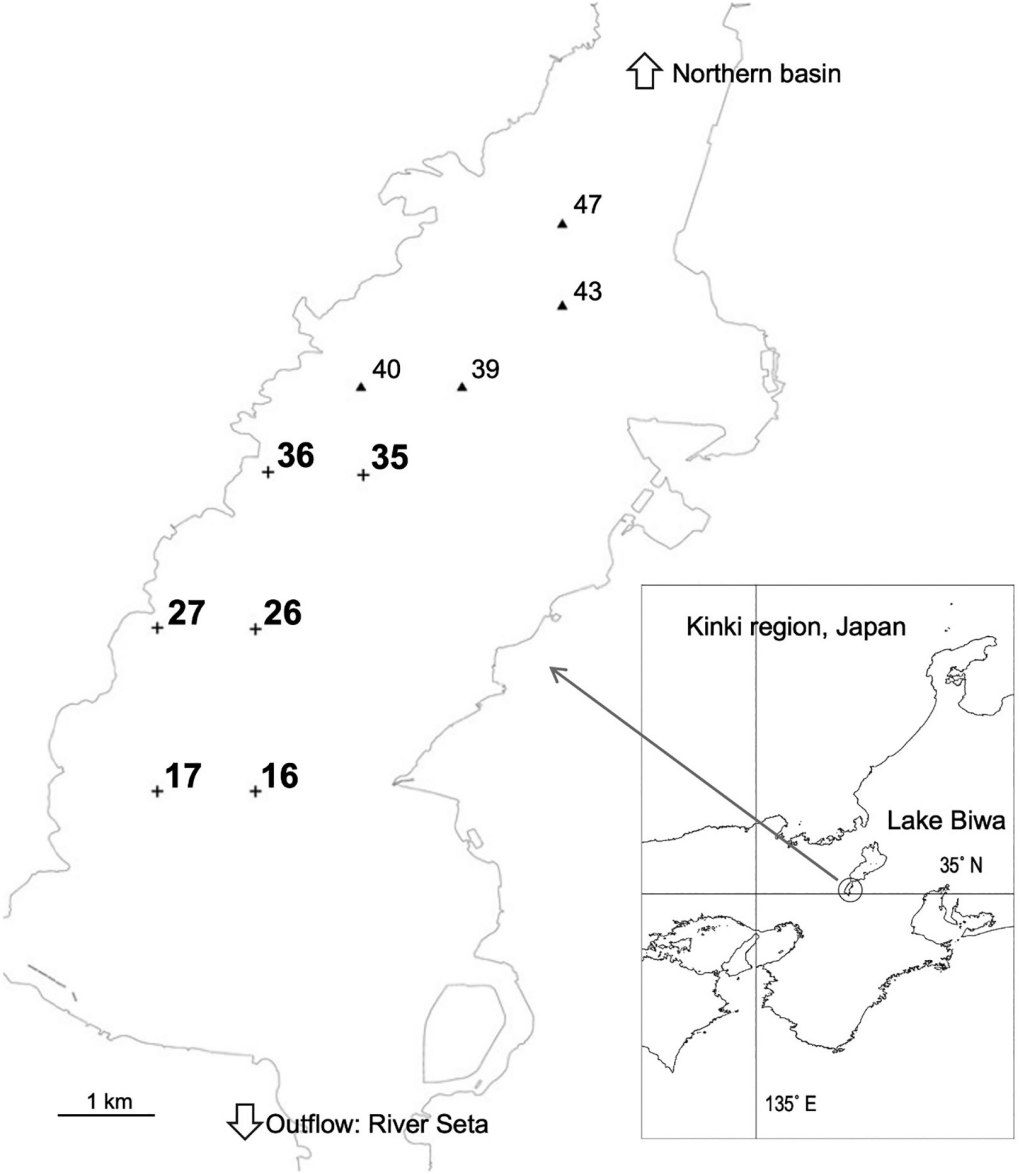
Location of the collection sites in the southern basin of Lake Biwa. Crosses with black letters indicate collection sites of macrobenthos in the submerged macrophyte (SM) zone, and crosses with gray letters indicate those in the benthic cyanobacterial (BC) zone. Triangles with italic letters are additional collection sites of macrophytes for stable‐isotope analysis. Site numbers are the same as those in Haga, Sakai, and Ishikawa (2019) and Ishikawa et al. (2019).

Many limnological studies have been conducted in the southern basin of Lake Biwa, particularly on the growth of SMs (e.g., Haga, Sakai, and Ishikawa 2019; Inoue, Kobayashi, and Nishino 2020; Ishikawa et al. 2019; Ishikawa, Inoue, and Hamabata 2020; Nakada et al. 2021). The abundance of benthic filamentous algae was measured for the first time by Haga, Sakai, and Ishikawa (2019). They measured the abundance of benthic filamentous algae, mostly composed of *M. wollei*, along with the abundances and species composition of SMs at 52 sites in the southern basin of Lake Biwa. The sampling sites for the present study were selected from among these sites. Six sites were selected for quantitative sampling of macrobenthos. Three sites, namely 17, 26, and 35, were located in the zone where the benthic filamentous cyanobacterium *M. wolle*i was dominant (BC zone), and the other three sites, namely 16, 27, and 36, were in the zone where SMs were dominant (SM zone) (Figure 1). Sampling was performed on June 5, 2017. The sampling date was set in early summer, when the growth of macrophytes, BC, and macrobenthos was expected to be high. Two bottom sediment samples were collected using an Ekman‐Birge grab sampler (15‐cm square in mouth) at each site and numbered as 16–1, 16–2, 17–1, 17–2, and so forth. The bottom depths of the collection sites were 3.7–4.9 m (Table 1). The lake bottom of the southern basin deepened steeply just off the shore to approximately 3 m, and then gradually to 6–7 m. Among the macrobenthos samples, *M. wollei* was collected at all three sites in the BC zone, but at a few, if any, sites in the SM zone. Macrophytes were collected from the two SM zone sites.

**TABLE 1:** Environmental variables at six sites in the southern basin of Lake Biwa. The benthic cyanobacterial (BC) zone is shaded.

Site	16	17	26	27	35	36
Depth (m)	4.5	4.6	4.6	3.7	4.2	4.9
Secchi transparency (cm)	120	122	155	156	252	183
Bottom temperature (°C)	21.8	22.1	21.7	22.0	21.5	22.1
Water temperature (°C)	22.1	22.3	22.3	22.2	21.3	22.3
Conductivity (mS/cm)	0.143	0.141	0.143	0.144	0.137	0.154
Chlorophyll *a* (μg/L)	4.95	3.62	5.09	6.37	6.30	14.80
Turbidity (FTU)	28.9	29.9	57.1	36.7	58.6	36.9
pH	7.80	8.04	8.01	8.01	8.60	8.10
Dissolved oxygen (mg/L)	7.57	7.75	7.76	7.81	8.53	7.97

At these sites, the bottom was mostly muddy, and the bottom sediment collected until a depth of approximately 5 cm was sieved using a NGG40 nylon mesh (470‐μm opening) bag from the collection boat. The samples were then transported to a laboratory on ice. Macrobenthic specimens were sorted from the samples using the naked eye and/or under a digital microscope (Leica DMS1000) at up to 60× magnification while being kept cool. A total of 197 specimens were collected, individually photographed, placed in a 1.5‐mL microcentrifuge tube, and frozen at −30°C.

Environmental variables at the study sites were measured at the same time as the benthos collection, at 8:00–10:00 a.m. (Table 1). These included water depth, Secchi‐disc transparency, bottom temperature, water temperature, conductivity, turbidity, chlorophyll‐*a* concentration, pH, and dissolved oxygen concentration. The latter six variables were measured at the bottom layer of the water column, using a water‐quality profiler (AAQ‐RINKO126; JFE Advantech Co., Japan). The profiler was maintained at approximately 25 cm above the bottom surface, as the measurement range of the optical sensor was 25 cm (JFE Advantech Co., personal communication). The bottom temperature was measured in the bottom sediment collected using an Ekman‐Birge grab.

### 2.2 DNA Extraction and Sequencing

Species were identified morphologically during sorting. The major taxonomic groups in this collection were chironomids, oligochaetes, amphipods, asellids, and leeches. Key morphological characteristics were obtained from Nihon Yusurika Kenkyu‐kai (2010) for chironomids, Ohtaka and Nishino (1995, 1999) for oligochaetes, and Tomikawa and Morino (2012) for amphipods. All specimens that had not been identified at the species level, and some specimens identified morphologically, were identified through DNA barcoding. In these cases, a part of the specimen body was dissected, or some amount of body tissue or fluid was absorbed onto a filter paper, and placed into 1.5‐ml microcentrifuge tubes for DNA extraction. We kept the other parts of the specimens intact as much as possible for reidentification.

DNA was extracted using the DNeasy Blood & Tissue Kit (Qiagen, Germany) following the manufacturer's protocol. PCR amplification was performed to extract DNA from the mitochondrial DNA COI region using a standard primer set (Folmer et al. 1994) and GoTaq Green MasterMix (Promega, U.S.). The PCR was an initial step of 95°C for 2 min, followed by 35 cycles of at 95°C for 30 s, 44°C for 45 s, and 72°C for 1 min, after which a final extension of 72°C was performed for 5 min. The annealing temperature was initially 55°C but later adjusted to 44°C, which followed the methods of Vivien et al. (2015). DNA amplification was confirmed using agarose gel electrophoresis.

Amplified DNA was sequenced using the BigDye DNA Terminator version 3.1 Cycle Sequencing Kit (Applied Biosystems, USA) and an ABI 3730 Genetic Analyzer (Applied Biosystems, USA). The resulting sequences were assembled using the MEGA11 software (Tamura, Stecher, and Kumar 2021).

In cases where the full‐length COI region was not precisely sequenced, the downstream region was sequenced using the primer set mlCOIintF (Leray et al. 2013) and HCO2198 (Folmer et al. 1994). All DNA sequences analyzed in this study were registered in the DNA Databank of Japan (https://www.ddbj.nig.ac.jp) under accession numbers LC671927–LC671971.

### 2.3 Molecular Species Delimitation

The COI DNA sequences of macrobenthos specimens that were not morphologically identified were identified using molecular species delimitation. They were compiled as haplotypes on the FaBox platform (Villesen 2007), and the haplotype sequences were aligned and read using BEAST2 (version 2.5; Bouckaert et al. 2019; Drummond and Rambaut 2007) to reconstruct a phylogenetic tree using a molecular clock model (strict clock). The best model for nucleotide substitution was selected as GTR + I + G, using jModelTest 2 (Darriba et al. 2012; Guindon and Gascuel 2003). Species were delimited on the phylogenetic tree using General Mixed Yule Coalescence (GMYC: Pons et al. 2006; Fujisawa and Barraclough 2013). The single threshold level (Fujisawa and Barraclough 2013) was adopted to segregate the speciation and coalescence bifurcations on the tree. For each species unit delimited, scientific names were determined as the best match on the BLAST search with ≥ 97% identity on the website of DNA Data Bank of Japan (https://www.ddbj.nig.ac.jp/services/blast.html) or EMBL‐EBI (Madeira et al. 2019). Of the 197 specimens collected, 192 were identified by morphological identification or molecular species delimitation of the full‐length COI DNA sequences. The remaining five specimens (four chironomids of *Chironomus plumosus* and one oligochaete of *Limnodrilus grandisetosus*) could not be sequenced precisely using the abovementioned method, likely due to the low extract‐DNA concentration or DNA degradation; therefore, the downstream section of the COI region (313 bp; Leray et al. 2013) was identified using a BLAST search.

### 2.4 Ordination of Macrobenthic Communities

Twelve samples (two samples from each of the six sites) of the macrobenthic community were classified by nonmetric multidimensional scaling (NMDS) using the R program package vegan (Oksanen et al. 2020) with the application of the similarity index of Chao, Shen, and Hwang (2006) in R version 4.1.2 (R Core Team 2019). The ordination was plotted in a two‐dimensional space. The quality of the configuration was determined based on the criteria with stress values (Zurr, Ieno, and Smith 2007). The samples were grouped using the cascadeKM function in the vegan package. The best grouping selected was also verified using PERMANOVA (1000 permutations) of the adonis function in the vegan package. Indicator species were selected for each group using the package labdsv (Roberts 2019) in which indicator species were defined as the most characteristic species of each group, found mostly in a single group of the typology, and present in the majority of the sites belonging to that group (Dufrene and Legendre 1997). The environmental variables measured at the study sites were projected on the ordination.

### 2.5 Carbon and Nitrogen Stable‐Isotope Analysis

To determine whether the macrobenthos assimilate organic carbon and nitrogen produced by benthic primary producers (BC and SMs), the δ^13^C (‰ vs. PDB) and δ^15^N (‰ vs. atmospheric N_2_) of the primary producers, macrobenthos, bottom plant debris, bottom surface sediment, particulate organic matter (POM), and fish were analyzed. Samples for stable‐isotope analysis were collected at sites 35 and 36 on June 4, 2018, for macrobenthos, *M. wollei*, and debris; from May to June 2018 for submerged plants; on June 10, 2019, for POM and surface bottom sediment; and on July 4, 2019, for fish. Because the used fish‐finder barely detected fish at these sites, they were caught with seine nets, with diver assistance, in the reed vegetation along the shoreline just west of the sites. Because of the low abundance of submerged plants at site 36, their specimens were collected by pulling a 50‐cm metallic bar armed with barbed wire on the lake bottom at the northern sites (sites 39, 40, 43, and 47: Haga, Sakai, and Ishikawa 2019; Ishikawa et al. 2019; Ishikawa, Inoue, and Hamabata 2020).

SMs (*Egeria densa*, *Elodea nuttallii*, *Hydrilla verticillata*, and *Potamogeton maackianus*) and *M. wollei* filaments were washed lightly to remove sediment. Macrobenthos (amphipods: *Crangonyx floridanus*, *Jesogammarus naritai*, and *Kamaka biwae*; an asellid: *Asellus hilgendorfii*; Tanypodinae chironomids; oligochaetes; and leeches) were analyzed as a whole body at a minimum dry weight of 0.15 mg to warrant precision, but smaller individuals were also analyzed as a single individual or in a lump (oligochaetes) (Takamura 2022) if larger individuals were not available. Macrobenthos samples, except for oligochaetes and leeches, were identified based on morphological characteristics confirmed through molecular species delimitation. For fish samples, omnivoro*is Lepomis macrochirus*, piscivorous *Micropterus salmoides*, epilithic algae feeding or zooplanktivorous *Plecoglossus altivelis* (Azuma 1973), piscivorous *Silurus asotus*, and omnivorous *Tridentiger brevispinis*, all of which are common in the southern basin of Lake Biwa were caught and muscle pieces were subsampled from the dorsal part of the body. Bottom plant debris was sieved from bottom sediments, using a 470‐μm mesh net during the benthos collection. POM in the water was collected on a Whatman GF/F glass fiber filter from a water sample collected with an acrylic tube sampler (4.8‐cm inner diameter, 1‐m long) in the middle layer of the water column. Bottom surface sediments were scooped from the surface 1‐cm layer of the grab sample. All fresh samples were freeze‐dried, and macrophyte, cyanobacterial, and fish samples were powdered. The carbonate contents in the POM, bottom plant debris, and bottom sediment samples were removed via washing or fumigating using hydrochloric acid (Jaschinski, Hansen, and Sommer 2008; Schlacher and Connolly 2014). Carbonate produced by crustaceans was removed from the asellid samples in the same way as above to determine how strongly such carbonate affected the δ^13^C values of crustaceans. For fish samples, fatty acids poor in nitrogen were removed via washing using a methanol‐chloroform mixture. All analyses were performed using a DELTA V Advantage mass spectrometer connected to a Flash EA 1112 elemental analyzer (Thermo Fisher Scientific, USA) at ≤ 0.1‰ precision.

To analyze the food web structure, we adopted the IsoWeb (Kadoya, Osada, and Takimoto 2012) model for isotopic data. From available data of both food and consumers, this model analyzes the entire food web together using the Bayesian simulation‐based method (MCMC) and quantifies the dietary proportions of each consumer category (species or species group). Based on the isotopic data and biological information on the food and consumers, we created a topological food web that reflected the presence or absence (1 or 0, respectively) of a predator–prey relationship between food and consumer, after which we introduced it into the model calculation.

## 3 Results

### 3.1 Macrobenthic Species Composition

**FIGURE 2:**
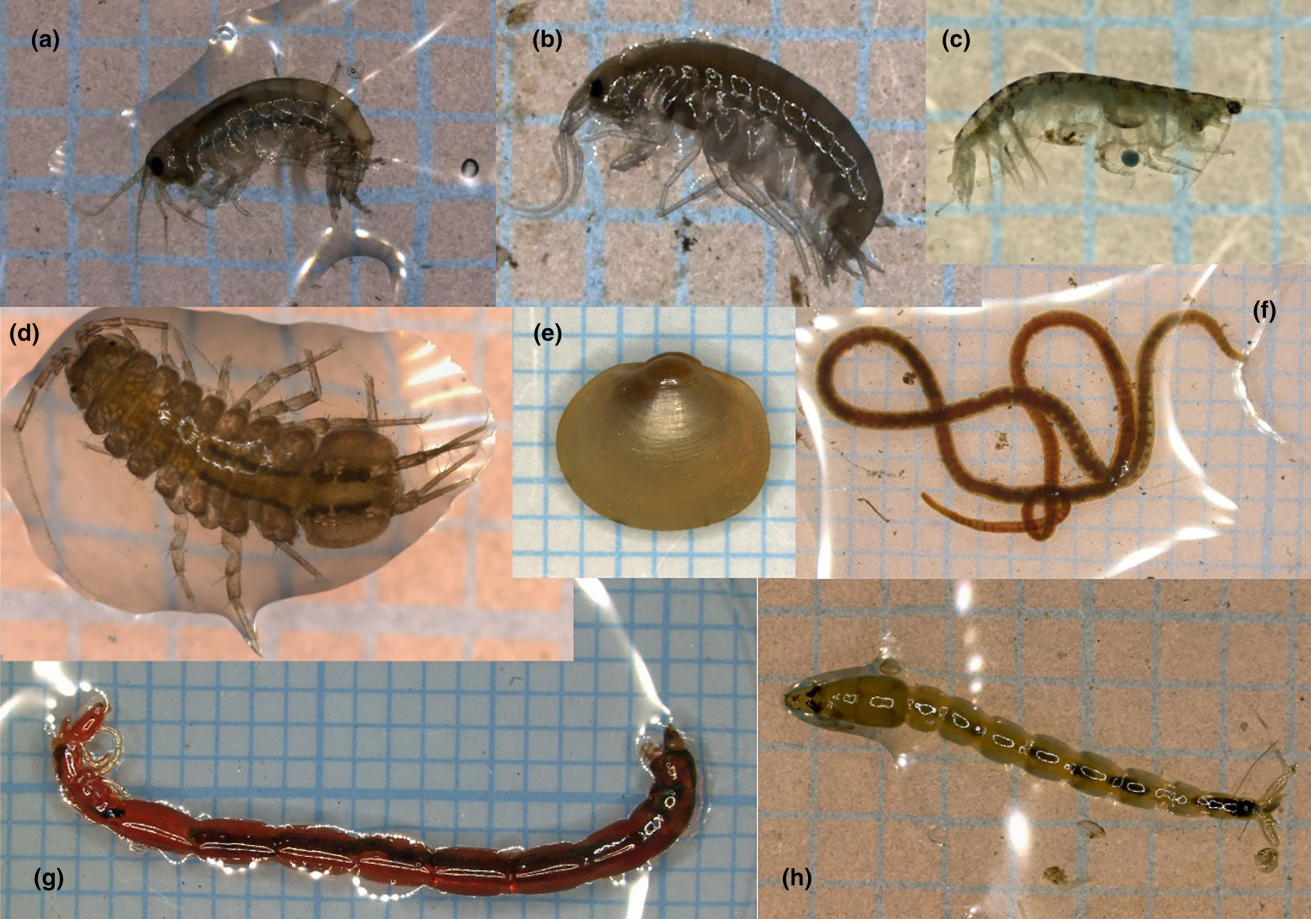
Some macrobenthic species collected in this study. (a) *Jesogammarus naritai*, (b) *Crangonyx floridanus*, (c) *Kamaka biwae*, (d) *Asellus hilgendorfii*, (e) *Sphaerium biwaense*, (f) *Limnodrilus grandisetosus*, (g) *Chironomus plumosus*, and (h) *Procladius choreus*. Specimens were photographed on 1‐mm grids.

**FIGURE 3:**
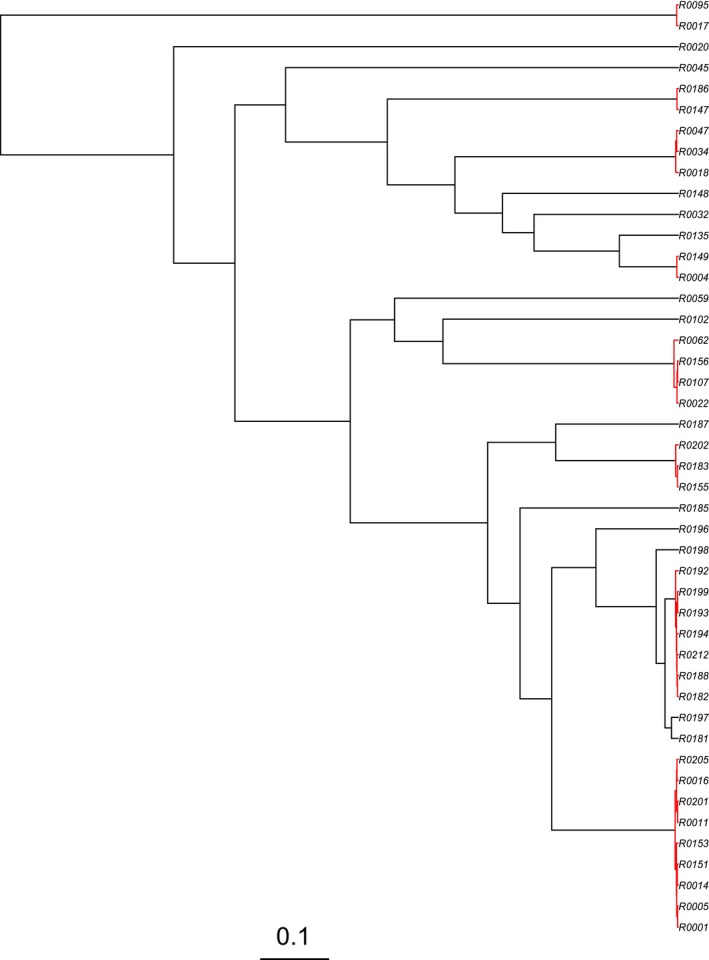
Phylogenetic tree of macrobenthic specimens with species delimitation using the general mixed yule coalescence (GMYC) method. Each red‐colored clade and singleton was delimited as a species, but three clades were actually identified as one species *M*. *tener* (see the text). The scale bar refers to a nucleotide substitution rate of 0.1. The specimen IDs of each species are shown in Table 2.

Eighteen species were identified (Figures 2 and 3; Table 2). For the chironomid *Microchironomus tener*, three taxa are delimited in Figure 3. In a previous study on pond chironomid communities (Takamura et al. 2021), this species was also delimited as three taxa, using the PTP method (Zhang et al. 2013), but as one taxon using the GMYC method, and it was consequently regarded as one species. Although the taxonomic status of this species requires further investigation, we followed the identification by Takamura et al. (2021).

**TABLE 2:** List of specimen IDs in Figure 3, and corresponding species names and species codes.

Specimen ID	Species name	Species code
R0017, R0095	*Sphaerium biwaense*	Sb
R0020	*Physella acuta*	Pa
R0045	*Sinotaia quadrata*	Sq
R0147, R0186	Hirudinea sp.	Hsp
R0018, R0034, R0047	*Limnodrilus grandisetosus*	Lg
R0148	*Bothrioneurum vejdovskyanum*	Bv
R0032	*Limnodrilus hoffmeisteri*	Lh
R0135	Oligochaeta sp.	Osp
R0004, R0149	*Branchiura sowerbyi*	Bs
R0059	*Asellus hilgendorfii*	Ah
R0102	*Crangonyx floridanus*	Cf
R0022, R0062, R0107, R0156	*Jesogammarus naritai*	Jn
R0187	*Procladius choreus*	Pc
R0155, R0183, R0202	*Psectrocladius yunoquartus*	Py
R0185	*Polypedilum masudai*	Pm
R0196	Chironomidae sp.	Csp
R0181, R0182, R0188, R0192, R0193, R0194, R0197, R0198, R0199, R0212	*Microchironomus tener*	Mt
R0001, R0005, R0011, R0014, R0016, R0151, R0153, R0201, R0205	*Chironomus plumosus*	Cp

Eighteen species comprised of 197 individuals were collected from 12 samples at six sites (Table 3). The collected specimens included 48 chironomids, 24 annelids, 10 mollusks, and 115 crustaceans. Initially, the sites appeared to be divided into two groups based on their species composition. One group was composed of sites 16, 27, and 36, and the other was composed of sites 17, 26, and 35. In the former group, chironomids, especially *C. plumosus* were dominant, whereas in the latter, asellid *A. hilgendorfii* and amphipods, especially *C. floridanus* were dominant.

**TABLE 3:** Species composition of macrobenthos in each of two samples from six sites in the southern basin of Lake Biwa. The benthic cyanobacterial (BC) zone is shaded. Cells filled with “—” indicate that no individuals were collected.

Site‐sample	16‐1	16‐2	17‐1	17‐2	26‐1	26‐2	27‐1	27‐2	35‐1	35‐2	36‐1	36‐2
**Chironomid**												
*Chironomus plumosus*	8	3	—	—	—	—	4	2	—	—	4	6
*Microchironomus tener*	—	—	—	—	—	—	—	—	—	—	4	11
*Polypedilum masudai*	—	—	—	—	—	—	—	—	—	—	1	—
*Psectrocladius yunoquartus*	—	—	—	—	—	—	—	—	—	1	1	1
*Procladius choreus*	—	—	—	—	—	—	—	—	—	—	1	—
Chironomidae sp.	—	—	—	—	—	—	—	—	—	—	—	1
**Oligochaete**												
*Limnodrilus grandisetosus*	—	1	1	4	3	2	—	—	—	3	—	—
*Limnodrilus hoffmeisteri*	—	—	1	—	—	—	—	—	—	—	—	—
*Branchiura sowerbyi*	1	1	—	—	—	—	2	—	—	1	—	—
*Bothrioneurum vejdovskyanum*	—	—	—	—	—	—	1	—	—	—	—	—
Oligochaeta sp.	—	—	—	—	—	1	—	—	—	—	—	—
Hirudinea sp.	—	—	—	—	—	—	1	—	—	—	1	—
**Mollusk**												
*Sphaerium biwaense*	—	1	—	1	3	1	—	—	—	—	—	—
*Physella acuta*	—	3	—	—	—	—	—	—	—	—	—	—
*Sinotaia quadrata*	—	—	1	—	—	—	—	—	—	—	—	—
*Asellus hilgendorfii*	—	—	7	35	5	8	—	—	—	9	—	—
**Amphipod**												
*Crangonyx floridanus*	3	—	9	13	1	8	—	—	1	9	—	—
*Jesogammarus naritai*	—	—	1	2	—	1	—	—	—	3	—	—

**FIGURE 4:**
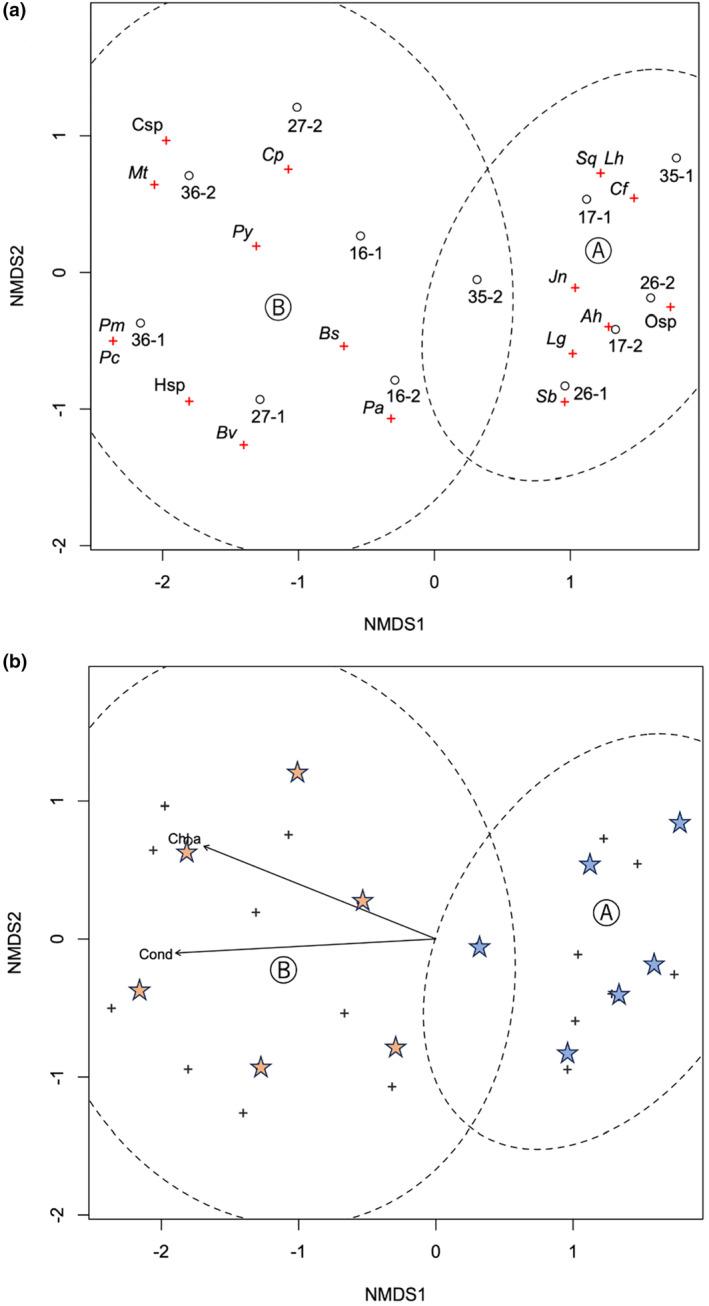
Nonmetric multidimensional scaling (NMDS) ordination plot of macrobenthic communities in the southern basin of Lake Biwa. (a) Open circles with black letters indicate community samples in the submerged macrophyte (SM) zone, and those with gray letters indicate those in the benthic cyanobacterial (BC) zone. Ellipses indicate confidence areas (95%) for two groups of communities. Crosses with the species code (Table 1) indicate species. (b) Ordination plot with environmental variables. Only significant variables (chlorophyll *a* and conductivity) at *p* < 0.05 are plotted. Stars indicate community samples.

The mulvariate NMDS analysis supported this trend. These zoobenthic samples were well‐configured in a two‐dimensional plot and classified into two groups (A and B: Figure 4a). A good configuration was indicated by the stress value of 0.070, according to the criteria (Zurr, Ieno, and Smith 2007). Group A included six samples from sites 17, 26, and 35, which were associated with the BC zone. Group B included six samples from sites 16, 27, and 36, which were associated with the SM zone. This grouping was regarded as most likely, usingthe Calinski criterion, as it showed a much larger value (17.7) than groupings of higher numbers (≤ 15.7). PERMANOVA also verified a significant difference between the two groups (*p* = 0.003). Group A contained three indicator species: the amphipod *C. floridanus* (*p* = 0.006), the asellid *A. hilgendorfii* (*p* = 0.019), and the oligochaete *L. grandisetosus* (*p* = 0.037). Group B consisted only one indicator species, *C. plumosus* (*p* = 0.006).

The environmental variables measured were similar at most sites (Table 1), but transparency was much higher at sites 35 and 36, conductivity and chlorophyll *a* were higher at site 36, turbidity was higher at sites 26 and 35, and pH and dissolved oxygen were higher at site 35. The chlorophyll‐*a* content and conductivity of the environmental variables were fitted on the ordination, with a significant correlation (*p* < 0.05, based on random permutations) (Figure 4b). These variables appeared to indicate a gradient of eutrophication and were associated with the distinction between Groups A and B. In addition, site 36 was characterized not only by higher concentrations of chlorophyll *a* concentration and higher conductivity but also by a richer chironomid fauna, among the Group B sites; therefore, this site may best represent the characteristics of this group.

### 3.2 Food Web Structure

**FIGURE 5:**
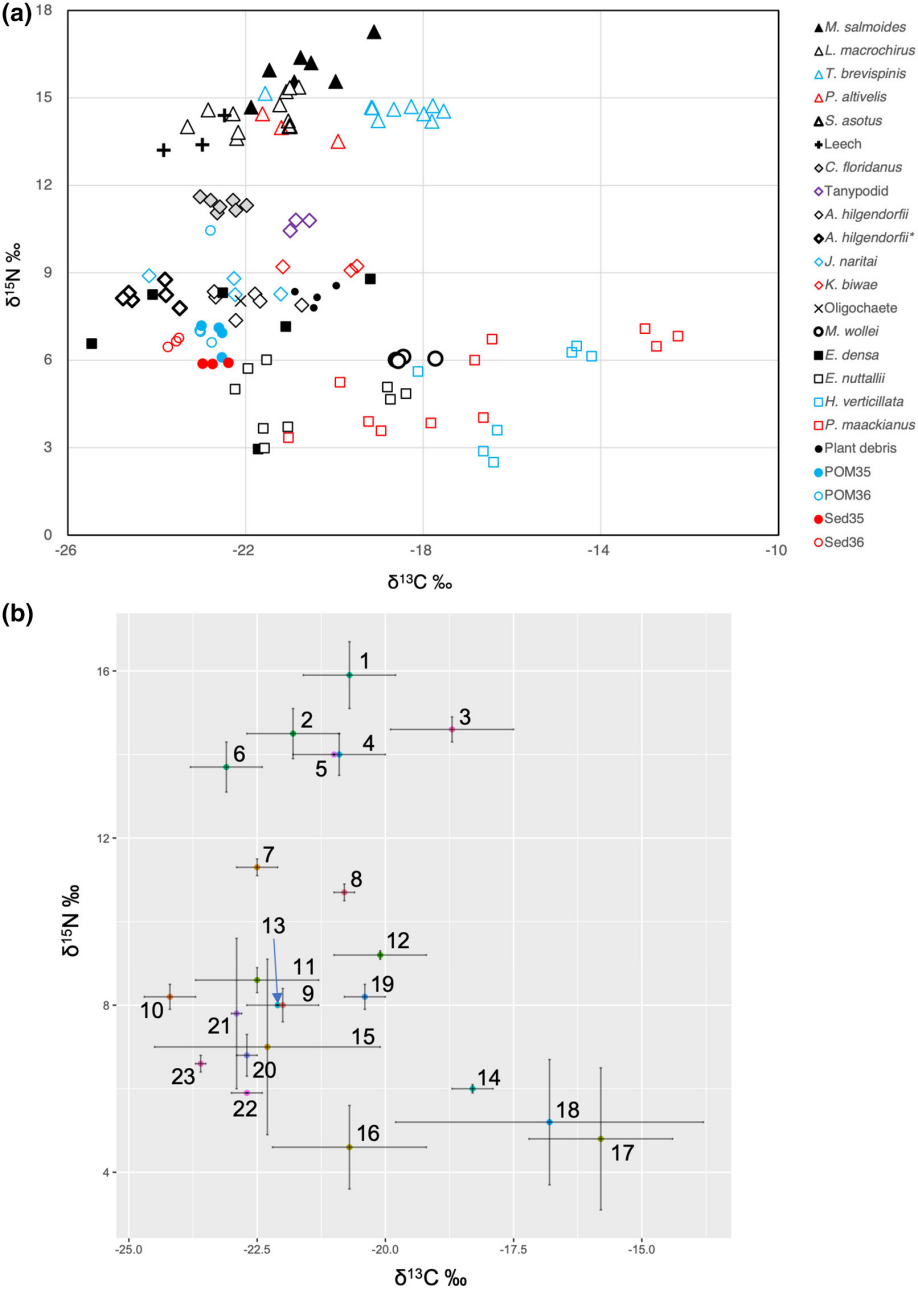
Dual isotope (δ^13^C and δ^15^N) plot of macrobenthos, fish, submerged macrophytes, *Microseira wolle*i, plant debris, bottom sediment, and POM from the southern basin of Lake Biwa. (a) Each point of macrobenthos and fish shows a value for an individual specimen, except for oligochaetes. Triangles: Fish, diamonds: Macrobenthos, and squares: Submerged macrophyte. Asellus hilgendorfii* were decarbonated using acid treatment. POM and bottom sediment (Sed) were from sites 35 (BC zone) and 36 (SM zone), respectively. (b) Mean δ^13^C and δ^15^N values with error bars (standard deviation) for each category of samples. Number labels are 1: *M. salmoides*, 2: *L. macrochirus*, 3: *T. brevispinis*, 4: *P. altivelis*, 5: *S. asotus*, 6: Leech, 7: *C. floridanus*, 8: Tanypodid, 9: *A. hilgendorfii*, 10: *A. hilgendorfii* decarbonated, 11: *J. naritai*, 12: *K. biwae*, 13: Oligochaete, 14: *M. wollei*, 15: *E. densa*, 16: *E. nuttallii*, 17: *H. verticillata*, 18: *P. maackianus*, 19: Plant debris, 20: POM35, 21: POM36, 22: Sed35, and 23: Sed36. Categories with a single specimen (*S. asotus* and oligochaete) are presented without error bars.

The δ^13^C and δ^15^N varied depending on the species and individuals (Figure 5). First, we noticed that the δ^13^C of *M. wollei* had low variability, while that of SMs ranged widely. The values of *M. wollei* were located at approximately −18‰. All macrobenthos and most fish samples had lower values of δ^13^C than this cyanobacterium.

The δ^13^C values of SMs ranged widely from −26‰ to −13‰. They were higher for *P. maackianus* and *H. verticillata* but lower for *E. nuttallii* and *E. densa*. The latter two species covered the range of δ^13^C for both macrobenthos and fish. The δ^13^C values of bottom sediment and POM were within the range of −24‰ to −22‰ and were closer to those of macrobenthos. The values of bottom plant debris were slightly higher.

The crustacean arthropods comprised three species and had δ^13^C values ranging from −25‰ to −19‰, and the values for *K. biwae* were relatively higher. The acid treatment applied to *A. hilgendorfii* lowered the mean δ^13^C value from −22.0‰ to −24.2‰, further deviating from those of *M. wollei*.

The δ^15^N values ranged from 2‰ to 18‰ (Figure 5). The values were mostly at the level of 8‰–9‰ for four macrobenthic taxa (*A. hilgendorfii*, *K. biwae*, *J. naritai*, and oligochaetes). Compared to them, δ^15^N values were approximately 3‰ higher (at the level of 11‰–12‰) for *C. floridanus* and Tanypodinae chironomids and much higher (at the level of 13‰–17‰) for fish and leeches. Assuming that the former macrobenthos taxa were primary consumers, and taking into account that the mean trophic fractionation of δ^15^N per trophic level was 3.4‰ (Post 2002), the latter macrobenthos and fish were carnivores or, simply, secondary or higher consumers.

The dietary proportions of food items for macrobenthos and fish analyzed using IsoWeb are listed in Table 4. *Microseira wollei* accounted for only approximately 10% of the diet of each species. POM and bottom surface sediment accounted for larger proportions of the diet of macrobenthos, in the range of 12%–15%, respectively, and the total proportion of two macrophyte species, *E. nuttallii* and *E. densa*, was 21%–29%.

**TABLE 4:** Dietary proportions of fish and macrobenthos quantified using the IsoWeb model. Cells for crustacean macrobenthos are shaded. Blank cells indicate that there is no predator–prey relationship supposed.

Food	Consumer																			
	*M. salmoides*		*S. asotus*		*L. macrochirus*		*T. brevispinis*		*Leech*		*C. floridanus*		*A. hilgendorfii*		*J. naritai*		*K. biwae*		Oligochaete	
	mean	SD																		
*L. macrochirus*	0.335	0.224	0.343	0.238	—	—	—	—	—	—	—	—	—	—	—	—	—	—	—	—
*T. brevispinis*	0.314	0.219	0.295	0.225	—	—	—	—	—	—	—	—	—	—	—	—	—	—	—	—
*P. altivelis*	0.352	0.229	0.363	0.241	—	—	—	—	—	—	—	—	—	—	—	—	—	—	—	—
Leech	—	—	—	—	0.170	0.126	0.157	0.125	—	—	—	—	—	—	—	—	—	—	—	—
*C. floridanus*	—	—	—	—	0.158	0.126	0.148	0.125	0.191	0.146	—	—	—	—	—	—	—	—	—	—
Tanypodid	—	—	—	—	0.149	0.125	0.162	0.130	0.169	0.139	0.109	0.093	—	—	—	—	—	—	—	—
*A. hilgendorfii*	—	—	—	—	0.128	0.110	0.122	0.111	0.157	0.132	0.119	0.103	—	—	—	—	—	—	—	—
*J. naritai*	—	—	—	—	0.132	0.112	0.131	0.116	0.169	0.143	0.116	0.102	—	—	—	—	—	—	—	—
*K. biwae*	—	—	—	—	0.130	0.111	0.149	0.127	0.151	0.130	0.104	0.094	—	—	—	—	—	—	—	—
Oligochaete	—	—	—	—	0.133	0.112	0.130	0.113	0.162	0.134	0.115	0.101	—	—	—	—	—	—	—	—
*M. wollei*	—	—	—	—	—	—	—	—	—	—	0.081	0.078	0.114	0.106	0.111	0.107	0.122	0.103	0.243	0.188
*E. densa*	—	—	—	—	—	—	—	—	—	—	0.120	0.106	0.144	0.123	0.145	0.125	0.126	0.106	—	—
*E. nuttalii*	—	—	—	—	—	—	—	—	—	—	0.092	0.086	0.147	0.125	0.142	0.120	0.128	0.108	—	—
*H. verticillata*	—	—	—	—	—	—	—	—	—	—	0.068	0.066	0.103	0.095	0.098	0.091	0.117	0.099	—	—
*P. maackianus*	—	—	—	—	—	—	—	—	—	—	0.076	0.072	0.109	0.100	0.105	0.096	0.122	0.104	—	—
Plant debris	—	—	—	—	—	—	—	—	—	—	—	—	0.106	0.097	0.110	0.100	0.122	0.103	0.211	0.173
POM	—	—	—	—	—	—	—	—	—	—	—	—	0.134	0.116	0.138	0.116	0.129	0.107	0.253	0.189
Bottom sediment	—	—	—	—	—	—	—	—	—	—	—	—	0.142	0.120	0.153	0.127	0.133	0.108	0.292	0.204

## 4 Discussion

The results of this study showed a clear contrast in the macrobenthic community between the BC zone and the SM zone. Alternate dominances of primary producers were also recorded at the same sites in September 2017 (Haga, Sakai, and Ishikawa 2019), 3 months after our macrobenthos collection, although *M. wollei* at one of the SM zone sites (site 16) was as abundant as that in the BC zone as they grew and covered the macrophytes.

### 4.1 Contrasting Dominance of Macrobenthos

Benthic crustaceans were dominant in the BC zone. Several studies have described a similar dominance in the mats of *M. wollei* (Gélinas et al. 2013; Hudon et al. 2012; Hudon, Sève, and Cattaneo 2014; Lévesque, Cattaneo, and Hudon 2015). The role of refuge from predation has been clearly indicated for *M. wollei* mats in North American lakes (Camacho and Thacker 2013; Hudon, Sève, and Cattaneo 2014; Lévesque, Cattaneo, and Hudon 2015), which may also be the case in Lake Biwa. However, the situation is somewhat different.

Amphipods and asellids were collected almost exclusively in the BC zone, but in Lake Saint‐Pierre, a fluvial lake of the St. Lawrence River (Quebec, Canada), amphipods were fairly abundant in the zone dominated by macrophytes as well as in the zone dominated by *M. wollei* (Hudon et al. 2012). In Lake Biwa, the peak macrophyte abundance occurred in 2002, 2007, and 2014, whereas the abundance decreased during the present study's period (Haga, Sakai, and Ishikawa 2019). Substantial changes in the trophic state that might have affected macrophyte abundance cannot be assumed for recent years, judging from the analyses of sediment cores (Hyodo et al. 2008) and pollution loads (Sato et al. 2016; Wada et al. 2020) of Lake Biwa. This decrease may have been due to phytoplankton blooms (Ishikawa et al. 2015), macrophyte herbivory (Carpenter and Lodge 1986), periphyton shading (Hilt et al. 2018), and/or mowing (Ishikawa et al. 2019). Although the abundances of amphipods and asellids were not reported at the time of peak macrophyte abundance, a large number of periphytic chironomids emerged from the lake water (Inoue, Kobayashi, and Nishino 2020), suggesting the presence of dense macrophyte stands as refuge from predation. In contrast, a lower amount of macrophytes was present in the SM zone in the present study; therefore, SMs may not have been abundant enough for amphipods and asellids to inhabit and avoid predation.

In contrast to the dominance of benthic crustaceans, chironomids were rare in the *M. wollei* zone. The low presence of chironomids in the BC zone was similar to that of Lake Teganuma (Takamura et al. 1989; Takamura and Iwakuma 1990), where the surface sediment of the lake bottom (approximately 1‐cm deep) was found to be highly anaerobic, but the surface bottom sediment was not anaerobic either in the SM or BC zones in supplementary measurements conducted in 2019 (Takamura 2022). In addition, diatoms, which are a probable food source for chironomids (Donahue et al. 2003; Furey et al. 2012; Kukuryk 2013) are common in the mats of Lake Biwa (Ohtsuka, Kitano, and Nakai 2018). Because most of the chironomid species, except for *P. yunoquartus*, found were sediment dwellers, the thick mat of *M. wollei* may have hindered their activity.

### 4.2 Potential of *M. wollei* as a Food Source for Consumers

The δ^13^C values of *M. wollei* were higher or equivalent to those of macrobenthos, demonstrating that *M. wollei* was not their major food source if the mean trophic fractionation of δ^13^C per trophic level was assumed to be approximately 0.4‰ (Post 2002). In addition, the dietary δ^13^C of amphipod crustaceans (*C. floridanus*, *J. naritai*, and *K. biwae*) may have been lower than those shown in Figure 5, as the acid treatment used to remove nondietary carbonate lowered the δ^13^C of the isopod crustacean *A. hilgendorfii* by as much as 2‰ (Figure 5). In contrast, the δ^13^C of SMs such as *E. nuttallii* and *E. densa* covered those of benthic invertebrates and fish, while varying widely between both species and individuals. The δ^13^C of SMs can vary widely depending on plant physiology and the environment (Keeley and Sandquist 1992; Takamura et al. 2007). As periphytic algae were not intentionally removed from the macrophyte samples for the stable‐isotope analysis, periphyton may have contributed to this variation. Periphytic algae are one among the preferred diets for benthic invertebrates such as chironomids, asellids, and amphipods (Jaschinski, Brepohl, and Sommer 2011; Jones and Waldron 2003). Although a detailed analysis of the discrimination of epiphytes from macrophytes is required, SMs attached by periphytic algae are likely to be one of the major food sources for macrobenthos in the southern basin of Lake Biwa, whereas BC are not. The results of the IsoWeb analysis generally support this conclusion, as the dietary proportion of macrobenthos in *M. wollei* was comparatively lower than that in the macrophytes, POM, and bottom sediment.

In studies reporting the dominance of benthic crustaceans in the mats of *M. wollei* (Gélinas et al. 2013; Hudon et al. 2012; Hudon, Sève, and Cattaneo 2014; Lévesque, Cattaneo, and Hudon 2015), of particular interest is how these crustaceans thrive on benthic cyanobacterium‐producing toxins such as saxitoxin. Some studies have reported that they may be heavily affected by the toxin, but they ingest the cyanobacteria (Gélinas et al. 2013) or even prefer it (Camacho and Thacker 2006; Lévesque, Cattaneo, and Hudon 2015). *Microseira wollei* is filamentous with discoid cells encased in a hard polysaccharide sheath, which deters feeding by amphipods (Camacho and Thacker 2006). However, some large amphipod species can consume *M. wollei* with strong mouthparts (Lévesque, Cattaneo, and Hudon 2015). In the southern basin of Lake Biwa, no toxic strains of *M. wollei* have been collected during surveys of the basin and genome sequencing (Yamaguchi, pers. com.; Yamaguchi, Suzuki, and Kawachi 2019; however, see Li 2018), but the stable‐isotope analysis showed that *M. wollei* was unlikely to be the prime food source for benthic crustaceans, other benthos, and fish (Figure 5). Furthermore, one of the amphipod species, *C. floridanus* appeared to be a secondary consumer and was rarely in a trophic position where it directly fed on *M. wollei*. Meanwhile, benthic crustaceans may prefer mats as a refuge as reported by Camacho and Thacker (2013) and Lévesque, Cattaneo, and Hudon (2015).

Although BC are not regarded as a major food source for macrobenthos, which is the case even in the BC zone, several studies have reported that they have a nonnegligible dietary value to freshwater invertebrates, along with a degree of harmfulness (Camacho and Thacker 2006; Gélinas et al. 2013; Hudon et al. 2012; Visconti et al. 2014). Presumably, they can be an indirect food source. Such an example is the case of the planktonic cyanobacterium *Microcystis*. These cyanobacteria often bloom in eutrophic lakes, and some of their strains produce toxins such as microcystin (Carmichael 1994). They are generally inedible to freshwater organisms such as zooplankton but become nutritious when decomposed (Luo, Liu, and Gulati 2015). For example, in Lake Kasumigaura, a Japanese eutrophic lake, zooplankton such as *Bosmina* and benthic chironomids rely on them in the warm or cool season when they are decomposed (Hanazato and Yasuno 1987a, 1987b; Hanazato 1991; Iwakuma and Yasuno 1987). Recent studies have shown that cyanobacteria are the main source of primary production in pelagic food webs (Briland et al. 2020; Eglite et al. 2019; Novotny et al. 2023; Suikkanen et al. 2021). *Microseira wollei* may be an indirect major food source for macrobenthos in lakes. The decomposing process may be microbial. Feeding by flagellates (Eglite et al. 2019) is also supposed.

As the δ^13^C of the bottom sediment and POM were close to those of macrobenthos, they were supposed to be taken by macrobenthos. They were mostly amorphous and were not analyzed further in terms of composition, but they appeared to be composed of phytoplankton and organic matter. This organic matter may have been decomposed macrophytes, periphytic algae or other types of organisms, and decomposing *M. wollei* might be included.

## 5 Conclusions

Before concluding this article, two things should be discussed concerning this study. First, in streams, rivers, and lakes, periphytic or planktonic primary producers show a wide range of δ^13^C due to variation in factors such as carbon sources, water movement, and diffusion resistance (Finlay 2001, 2004; Lammers, Reichart, and Middelburg 2017; Schindler et al. 1997). In this study, *M. wollei* showed a narrow range of variation in δ^13^C, higher than those of consumer organisms, but in Lake Saint‐Pierre, it showed mean δ^13^C values less than 24‰ (Hudon et al. 2012). *Microseira wollei* might have had a wider δ^13^C variation and matched with macrobenthos in this regard.

Second, a theoretical study indicated that periphyton on lake bottoms, which can dominate primary production in shallow clear‐water lakes, show weak resilience due to low light availability (Genkai‐Kato et al. 2012). However, the adaptation to low light intensity of *M. wollei* (Speziale, Turner, and Dyck 1991) is likely to sustain its benthic growth. In addition, the filaments of *M. wollei* are known to not be easily degradable under the anaerobic conditions of water (Doyle and Smart 1998). *Microseira wollei* is likely present throughout the year at the bottom of the southern basin of Lake Biwa (Takamura 2022), and these findings may help explain how these cyanobacteria grow and decompose, though their dynamics remain unclear.

This study is too short a period to address the long‐term dynamics of benthic communities in the southern basin of Lake Biwa and lacks comprehensive measures of food web analysis. Therefore, long‐term monitoring and species‐level food analysis should be performed for consumer organisms, including macrobenthos.

## Author Contributions

**Kenzi Takamura:** conceptualization (lead), data curation (lead), formal analysis (lead), funding acquisition (lead), investigation (lead), methodology (equal), project administration (lead), writing – original draft (lead), writing – review and editing (lead). **Natsuko I. Kondo:** data curation (supporting), methodology (equal), writing – original draft (supporting). **Nobuyoshi Nakajima:** data curation (supporting), methodology (equal).

## Conflicts of Interest

The authors declare no conflicts of interest.

## Data Availability

The datasets generated during the current study are included in this published article and/or in the NIES repository (https://doi.org/10.17595/20221118.001).
